# Tracking Cell Recruitment and Behavior within the Tumor Microenvironment Using Advanced Intravital Imaging Approaches

**DOI:** 10.3390/cells7070069

**Published:** 2018-07-03

**Authors:** Madison Turk, Victor Naumenko, Douglas J. Mahoney, Craig N. Jenne

**Affiliations:** 1 Department of Microbiology, Immunology and Infectious Diseases, Faculty of Medicine, University of Calgary, Calgary, AB T2N 4N1, Canada; madison.turk1@ucalgary.ca (M.T.); naumenko.vict@gmail.com (V.N.); 2 National University of Science and Technology “MISIS”, Leninskiy Prospect 4, 119991 Moscow, Russia; 3 Alberta Children’s Hospital Research Institute, Calgary, AB T2N 4N1, Canada; 4 Arnie Charbonneau Cancer Institute, Calgary, AB T2N 4N1, Canada; 5 Department of Biochemistry and Molecular Biology, Faculty of Medicine, University of Calgary, Calgary, AB T2N 4N1, Canada; 6 Department of Critical Care Medicine, Faculty of Medicine, University of Calgary, Calgary, AB T2N 4N1, Canada

**Keywords:** intravital, cancer, vasculature, trafficking, leukocytes, imaging

## Abstract

Recent advances in imaging technology have made it possible to track cellular recruitment and behavior within the vasculature of living animals in real-time. Using approaches such as resonant scanning confocal and multiphoton intravital microscopy (IVM), we are now able to observe cells within the intact tumor microenvironment of a mouse. We are able to follow these cells for extended periods of time (hours) and can characterize how specific cell types (T cells, neutrophils, monocytes) interact with the tumor vasculature and cancer cells. This approach provides greater insight into specific cellular behaviors and cell–cell interactions than conventional techniques such as histology and flow cytometry. In this report, we describe the surgical preparation of animals to expose the tumor and both resonant scanning confocal and multiphoton imaging approaches used to track leukocyte recruitment, adhesion, and behavior within the tumor microenvironment. We present techniques for the measurement and quantification of leukocyte behavior within the bloodstream and tumor interstitium. The use of IVM to study leukocyte behavior within the tumor microenvironment provides key information not attainable with other approaches, that will help shape the development of better, more effective anticancer drugs and therapeutic approaches.

## 1. Introduction

For more than 150 years, researchers have used microscopes to look into the tissues of living animals in an effort to better understand how blood flows, how cells interact, and how tissues function. From the pioneering work of Hall and Waller, who examined the outstretched tongue of a frog, to the seminal studies of Cohnheim, which helped establish the concept that white blood cells are recruited from the bloodstream through the vessel wall and into neighboring tissues, we have gained a truly functional understanding of the interrelationship between blood, leukocytes, and tissues [1,2,3]. This approach of “intravital” microscopy (IVM) provides a number of advantages over the conventional histological examination of fixed or excised tissue samples. Most importantly, IVM allows for the study of tissue physiology and cellular behavior within the intact tissues of living animals. Whereas other approaches, such as flow cytometry, can provide insight into the cellular composition of a tissue, they only represent a single snapshot in time, and it is difficult to track cellular behavior over time using these approaches. Moreover, histology, flow cytometry, protein, and gene expression assays do little to shed light on the dynamics of vascular function, mechanisms of cell recruitment, cell–cell interactions and behavior within a tissue environment.

While IVM originally began as simple light microscopy, tracking unlabeled cells in thin, translucent tissues [1,2,3,4], the technique has continued to evolve, embracing ever-advancing imaging technologies. Two decades ago, IVM utilized single fluorophores and mercury lamp-illuminated fluorescent microscopy [5]. Today, IVM typically involves multiple fluorescent markers, laser-based excitation light sources, and advanced fluorescence detection systems. Although many IVM imaging platforms exist, for discussion purposes they can be generally grouped into three categories: spinning-disk confocal, laser scanning confocal, and multi-photon. Each imaging modality has its own advantages and disadvantages. Briefly, whereas conventional spinning-disk confocal imaging is, in large part, rather straightforward, allows for rapid image acquisition, and is very forgiving with respect to tissue movement, it tends to sacrifice sensitivity, flexibility in fluorophore selection, and image resolution. In contrast, conventional laser scanning confocal microscopy yields images of higher resolution and generally provides greater flexibility in fluorophore selection (due to tunable filters or spectral unmixing); however, this imaging modality often has a much slower image acquisition capability. This problem has, for the most part, been overcome in recent years with the advent of resonant scanning confocal microscopy, a technique that allows for extremely rapid image acquisition (30 frames per second). Of the three most common IVM platforms, multiphoton imaging provides the highest resolution and superior imaging depth but tends to be most restrictive in the number of fluorophores that are able to be simultaneously imaged because of the cost of the multiphoton pulse lasers required for fluorophore excitation (i.e., all fluorophores used must excite at a single laser wavelength, or multiple lasers are required). In reality, choosing the correct imaging platform often boils down to a determination of what specifically you are trying to image.

Although originally developed to characterize blood flow and leukocyte recruitment, IVM has more recently been employed to study a vast array of physiological and pathological processes. With this approach, researchers have mapped cellular dynamics within the lymph node germinal centers [6], pathogen clearance from the blood [7], wound repair [8], embryonic development [9,10], anti-viral immunity [11,12], coagulation [13], and angiogenesis [14]. Importantly, IVM has been used to identify a number of key physiological processes that had previously not been observed using conventional histological or flow cytometric approaches, including reverse transmigration of neutrophils out of tissues and back into the bloodstream [15], cellular plasticity within sites of tissue repair [16], necrotaxis [17], antigenic trafficking within lymphoid tissues [18], and cellular “swarming” [19].

IVM has also been utilized to study cancer, providing insight into tumor development [20,21,22], metastasis [23,24,25], and interactions with the immune system [21,26], and has helped contribute to our evolving understanding that “cancer” is not simply a condition of dysregulated cell proliferation and death, but rather often is comprised of a complex tumor microenvironment (TME) consisting of cancer cells, neovasculature, immune cells, supporting cells (cancer-associated fibroblasts), and extracellular matrix [27,28]. IVM allows us to “see” cells and their behavior within intact tissues and to track these cells and their effects on the tissue microenvironment over time. Indeed, recent advancements in IVM technology now allow us to study the TME in great detail, tracking, in real-time, how cytotoxic lymphocytes and myeloid cells interact with the tumor vasculature, how these cells egress out of the vessels and into the tumor proper, and how these cells move through the TME to interact with tumor cells. These observations are especially important for studying cancer immunotherapy. Tumor response to immunotherapy is often dependent on the ability of the immune system to detect cancer cells and to send specialized “killer” cytotoxic lymphocytes to a specific location. By “watching” these processes in the living animal, we can gain insight into potential “bottlenecks” (e.g., cell recruitment to the tumor, extravasation, movement through the tumor interstitium, etc.) and directly assay the effects of a given immunotherapy in vivo (e.g., adoptive T cell transfer [29], oncolytic viral (OV) treatment or infection [30], checkpoint inhibitors [31]). Importantly, the effects observed can be placed into context within the overall TME (i.e., increased leukocyte recruitment may not necessarily result in enhanced tumor clearance because of a lack of interaction between the leukocytes and cancer cells). Collectively, these features add significantly to the existing armamentarium of methods and techniques being used to study the interactions between immunotherapies, immune cells, and the complex TME. In the work below, we describe how IVM can be used to characterize the tumor vasculature, track leukocyte recruitment, and map cell movement through the TME.

## 2. Materials and Methods

### 2.1. Animals

BALB/c mice were purchased from Jackson Laboratories (Bar Harbor, ME, USA) and C57BL/6 mice were purchased from Charles River Laboratories (Montreal, QC, Canada). The mice used in this study were 7–10 weeks of age and were maintained in a specific pathogen-free environment at the University of Calgary Animal Resource Centre. All experimental animal protocols were approved by the University of Calgary Animal Care Committee and were in compliance with the guidelines from the Canadian Council for Animal Care (Protocol number AC15-0081).

### 2.2. Antibodies and Dyes

Antibodies used for imaging were purchased from BD Biosciences (San Jose, CA, USA) or Thermo Fisher Scientific (Waltham, MA, USA). Labelling of platelets and natural killer (NK) cells was achieved using phycoerythrin (PE)-conjugated anti-mouse CD49b (clone HMα2). Neutrophils were labelled with Brilliant Violet 421-conjugated anti-mouse Ly6G (clone 1A8). CD8+ cells were labelled with eFluor 660-conjugated anti-mouse CD8α (clone 53-6.7). Endothelial cells were labelled using PE-conjugated anti-mouse CD31 (clone 390). Typically, 1–3 ug of each labelling antibody was injected intravenously 10 min before imaging to ensure maximal labelling. Vasculature and perfusion were visualized using either fluorescein (FITC)-conjugate albumin or Qtracker 655 Vascular Label (Thermo Fisher Scientific).

### 2.3. Cell Culture

A frozen aliquot (1 × 10^6^ cells/mL of RPMI-1640 + 10% fetal bovine serum [FBS], 5% DMSO) of CT-26 colon carcinoma cells (JC Bell—Ottawa) or M3-9-M rhabdomyosarcoma (RMS) cells (Crystal MacKall—Stanford) were thawed in a 37 °C water bath, and 1 mL of cell suspension was added to 10 mL of RPMI 1640 media with 10% FBS. The cells were centrifuged to yield a cell pellet (800 *g* for 5 min at 4 °C), the supernatant was discarded, and the cells were resuspended in 10 mL of RPMI 1640 + 10% FBS. The cells were plated in a 10 cm petri dish and incubated at 37 °C for 1–2 days until confluent. Once confluent, the cells were lifted using trypsin (0.25%) + EDTA (0.913 mM) and split to an optimal plating density (~1–5 × 10^6^ cells/10 cm dish). The cells were passaged the day before injection.

### 2.4. Preparing Cells for Tumour Implantation

The tumor cells were lifted with trypsin (0.25%) + EDTA (0.913 mM), resuspended in 10 mL of RPMI + 10% FBS, and transferred to a 50 mL centrifuge tube. The cells were pelleted (800 *g* for 5 min at 4 °C), the supernatant was discarded, and the cells were resuspended in phosphate-buffered saline [PBS] at a concentration of 2 × 10^7^ cells/mL.

### 2.5. Tumor Implantation

The animals were restrained by hand or with an adapted 50 mL centrifuge tube. For subcutaneous tumors, the posterior flank of the animal was shaved to remove the fur, improving the visualization of the injection site, and washed with 70% ethanol. An aliquot of 2 × 10^7^ CT-26 cells was injected subcutaneously into BALB/c mice in a 50 µL volume, using a 30 ½ G needle and a 0.3 cc syringe. The tumors were allowed to establish for approximately 10 days before imaging. Alternatively, for intramuscular RMS tumors in C57BL/6 mice, the animal was restrained, a leg stabilized, and 2 × 10^5^ M3-9-M cells, in 50 µL of PBS, were injected into the gastrocnemius muscle at a location 1 mm above the base of the muscle. Again, the tumors were given approximately 10 days to establish before imaging. In some cases, the animals received an i.v. injection of fluorescently labelled vesicular stomatitis virus carrying a green fluorescent protein reporter gene (VSV^ΔM51^-GFP; 5 × 10^8^ plaque forming units) either 6 h prior to imaging or during the imaging process (i.e., imaging of viral delivery).

### 2.6. Surgical Preparation of Subcutaneous Tumours

**Figure 1:**
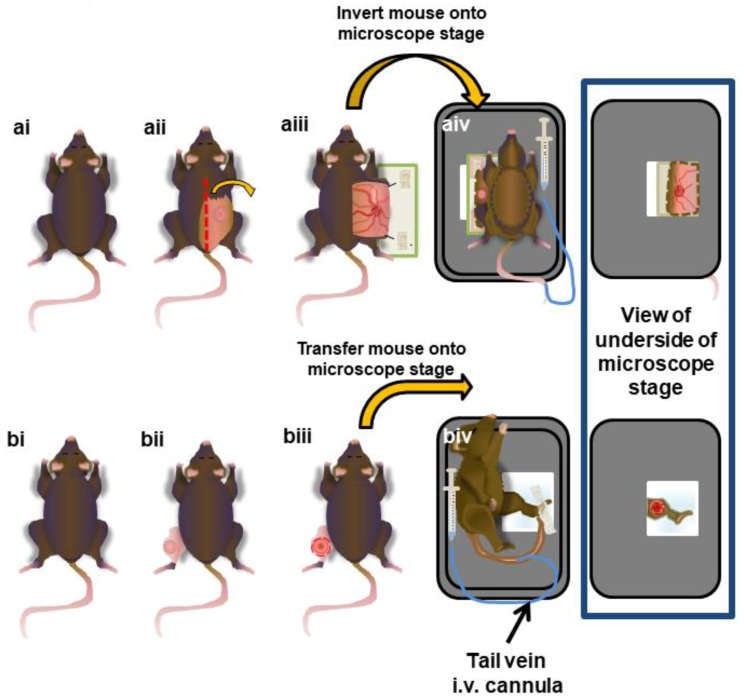
Surgical preparation of subcutaneous and intramuscular tumors for intravital microscopy (IVM) imaging. The mice were injected with tumor cells either subcutaneously on their flank (**a**) or intramuscularly in the gastrocnemius of the leg (**b**). After approximately 10 days, the tumors were exposed (**aii**,**bii**), tissue movement was surgically stabilized (**aiii**,**biii**), and the mouse was inverted and placed onto a heated (37 °C) microscope stage (**aiv**,**biv**). An i.v. cannula was inserted into the tail vein to provide anesthetic when necessary throughout the imaging procedure.

The animals were prepared as previously described [32]. Briefly, the mice were anaesthetized using an intraperitoneal injection of xylazine (10 µg/g) and ketamine (200 µg/g), and a venous catheter was inserted in the tail vein to allow the administration of labelling antibodies and dyes and the maintenance of the anesthetic. The mice were monitored throughout all surgical and imaging procedures for the depth of anesthesia. The mice were positioned on their abdomens on a heated pad (37 °C) and secured in place with surgical tape. Ethanol and sterile mineral oil were used to saturate the dorsal area to limit contamination of the surgical and imaging sites with fur. An incision was made from the base of the tail, just lateral to the spine, continuing up to the neckline on the side of animals with a tumor. The skin was lifted away from the body, reflected laterally, and the overlying fascia layer was removed. Two sutures were placed along the cut border of the skin flap to allow it to be stretched out and secured to a blank microscope slide. The animals were inverted and placed on their back on a heated microscope stage (37 °C), allowing the skin flap with the tumor to be extended over the imaging window, and the stage was then transferred to the inverted microscope. Surgeries are outlined in Figure 1a.

### 2.7. Surgical Preparation of Intramuscular Tumors

Similar to the preparation of subcutaneous tumors, the mouse was anesthetized, and the tail vein was cannulated. The tumor-bearing leg was extended, and a small incision was made in the skin located directly over the tumor mass. The skin layer was slowly removed, with care taken not to damage any major vessels. Once a window of skin was removed, the outer connective tissue layer was carefully removed, providing better exposure to the tumor vasculature for imaging. PBS was applied to the tumor every 10 min to maintain tissue moisture, and the animal was set in a lateral position on a heated microscope stage so that the tumor was located directly on the cover glass. The leg was gently secured in place with tape, with care taken not to block or restrict the blood flow. The stage was transferred to the microscope (Figure 1b).

### 2.8. IVM Imaging

IVM was performed using a Leica SP8 inverted microscope (Leica Microsystems, Concord, ON, Canada), outfitted with four excitation lasers (405-, 488-, 552-, and 638-nm), an 8 kHz tandem scan head, tunable emission filters, and spectral detectors (conventional photomultiplier tubes [PMTs] and hybrid HyD detectors). The typical imaging parameters for representative fluorophores were as follows: Brilliant Violet 421, excitation = 405 nm, detection = PMT with the tunable filter opened from 401 nm → 443 nm; Qtracker 655, excitation = 405 nm, detection = HyD with the tunable filter opened from 647 nm → 678 nm; FITC, excitation = 488 nm, detection = HyD with the tunable filter opened from 498 nm → 525 nm; GFP, excitation = 488 nm, detection = HyD with the tunable filter opened from 500 nm → 519 nm; PE, excitation = 552 nm, detection = PMT with the tunable filter opened from 566 nm → 595 nm; RFP, excitation = 522 nm, detection = PMT with the tunable filter opened from 585 nm → 627 nm; eFluor 660, excitation = 638 nm, detection = HyD with the tunable filter opened from 647 nm → 667 nm. Additionally, the system was equipped with a tunable multiphoton (MP) laser (700–1040 nm) (Newport Corporation, Irvine, CA, USA) and external PMT and HyD detectors (Leica) for deeper imaging of tissues (up to 800 µm). External PMT and HyD detectors have a shorter light path, reducing light scattering and allowing for imaging more deeply into tissues, and capture the emitted light without the need for a pinhole, allowing the collection of all emitted light and increasing the overall imaging sensitivity. Four-color multiphoton imaging can be achieved using an excitation wavelength of 980 nm and an emission filter set (560 nm dichroic mirror) that splits the emission fluorescence into two light paths. Light path 1 was comprised of a 495 nm dichroic and a 460/50 nm bandpass filter for BV421 together with a 525/50 nm bandpass filter for Alexa 488. Light path 2 was comprised of a 620 nm dichroic and a 575/15 nm bandpass filter for PE together with a 661/20 nm bandpass filter for Quantum Dot 655. Both confocal and multiphoton imaging utilized a 25× water immersion lens (HC FLUOTAR L 25×/0.95 W VISIR). This system was driven by, and data were recorded using, the LasX image acquisition software (Leica). In the event that fluorophores had overlapping emission spectra, sequential imaging was employed to collect data from each excitation laser independently.

### 2.9. Cell Tracking Analysis

Two approaches to cell tracking were used, as each software package has its own advantages and limitations. Cells within the vessel were tracked using Fiji package of ImageJ (NIH) [33] and the Manual Tracking plug-in. This was a suitable approach to tracking fast-moving cells in large vessels. Automated tracking software (such as the module in LasX) can be problematic for tracking single cells between frames at high velocities. This is due to the method of image acquisition, whereby videos were captured at 6 frames/min (to minimize photo-bleaching of the labels), creating discontinuous movies. Slower moving, interstitial, crawling, and extravasated cells were quantified and characterized efficiently using the cell-tracking modules within the LasX image acquisition software. Rolling cells were defined as cells moving slower than the flow of circulating platelets. Adherent cells were defined as cells remaining stationary for more than 30 s. Intravascular and extravascular cells were determined to be outside the vasculature by staining for endothelial cells (anti-mouse CD31). Meandering Index refers to the displacement of a cell in relation to the total distance it traveled (displacement/distance traveled). Typically, sample movement is not conducive to volume reconstructions and cell trafficking measurements. As such, individual surgical preparations with marked movement are either repositioned and re-secured to the microscope stage (hopefully eliminating the visualized movement) or excluded from 3D volume reconstruction analysis. With respect to cell dynamics, most cell-tracking platforms, such as the Leica software used in this study and the Fiji plugin package for ImageJ (“Manual Drift Correction” plugin for 2D imaging and the “Correct 3D Drift” plugin for 3D reconstructed images) [34], have movement correction functions whereby a specific region in the image is identified and registered. This registered point serves as a landmark, and cell movement is tracked relative to this point, ensuring accurate measurements of velocity, displacement, and distance travelled.

## 3. Results

IVM is an effective tool for assessing TME organization and cellular behavior within it. Through a combination of fluorescently labelled antibodies, fluorophore-conjugated proteins, and cells or viruses expressing or carrying fluorescent reporter protein genes (green fluorescent protein [GFP], red fluorescent protein [RFP]) it is possible to directly visualize and track multiple cell populations and anatomical structures within a tumor. Additionally, the use of multiphoton imaging allows the visualization of higher order protein structures, such as collagen, because of a natural phenomenon known as second-harmonic generation [35]. Initially, we used these techniques to assess the overall TME.

### 3.1. Multimodal IVM Imaging of the TME

**Figure 2:**
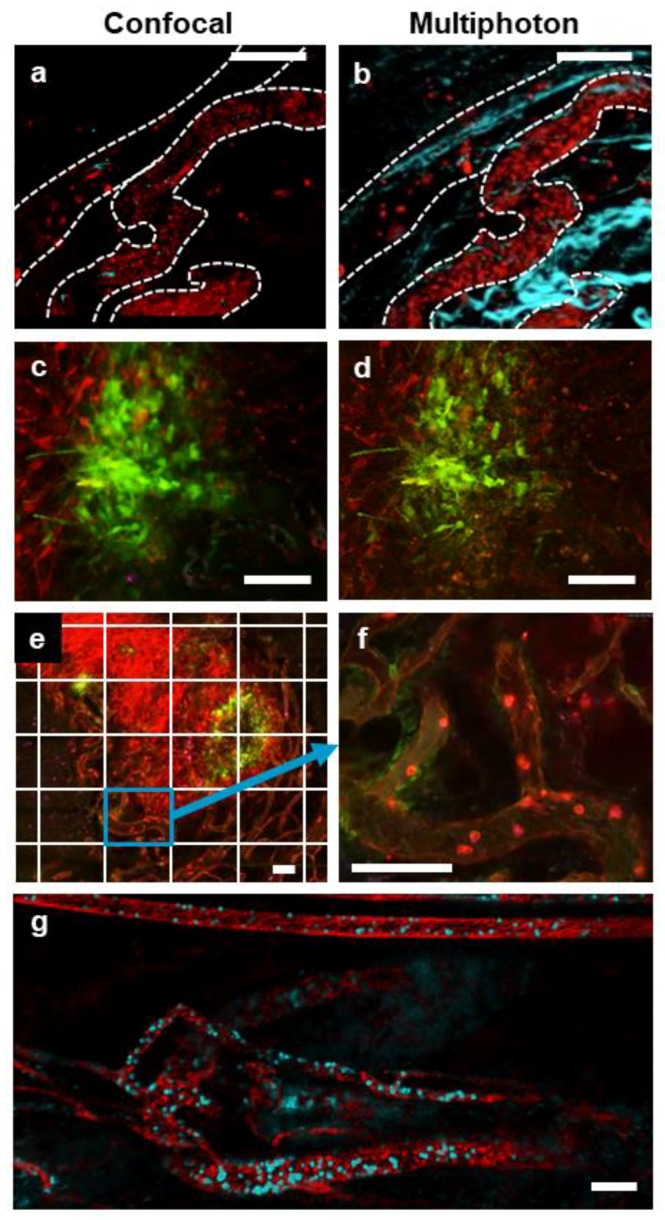
IVM of tumor vasculature acquired with resonant scanning confocal or multiphoton imaging modalities. Representative images of a single field of view showing a subcutaneous CT-26 tumor vasculature (red; phycoerythrin (PE)-conjugated anti-CD31 and PE-conjugated anti-CD49b, blood vessels are denoted by a dotted white outline) captured with either confocal (**a**) or multiphoton (**b**) imaging (collagen appears blue as a result of second-harmonic generation). In the confocal image (**a**), some CD8+ cells (blue; eFluor 660-conjugated anti-CD8α) are visible; however, these cells are not seen in the multiphoton image (**b**) because of a lack of fluorophore stimulation by the single multiphoton excitation wavelength used in this imaging. Confocal (**c**) and multiphoton (**d**) imaging of a subcutaneous CT-26 tumor (red) infected with VSV that is transgenic for GFP (VSV^ΔM51-GFP^; green). A stitched, tile scan image (**e**) of an entire subcutaneous CT-26 tumor (bright red) demonstrating localized VSV infection (green) was captured using resonant scanning confocal imaging. Note each tiled image (outlined by the white grid) was captured as a high-resolution video (**f**) allowing for later analysis at the cellular level (neutrophils in bright red, endothelium in dim red, i.v.-delivered eFluor 660-labelled VSV^ΔM51-GFP^ in blue). High-resolution 4D movies of stitched images (**g**) of the tumor vasculature of a rhabdomyosarcoma within the gastrocnemius muscle (red; PE-conjugated anti-CD31 and PE-conjugated anti-CD49b), containing neutrophils (cyan; BV421-conjugated anti-Ly6G) captured using multiphoton microscopy. The white scale bar represents 100 µm. Images in (**a**,**c**,**e**,**f**) were captured using resonant scanning confocal microscopy, whereas images in (**b**,**d**,**g**) were captured using resonant scanning multiphoton microscopy.

Subcutaneous and intramuscular tumors were imaged using both resonant-scanning confocal and multiphoton IVM. Imaging a single, common field of view, both modalities allow for a clear visualization of tumour-associated vasculature and leukocyte populations within the vessels and tissue (Figure 2a,b, Video S1). The addition of markers for both endothelium (anti-CD31) and platelets (anti-CD49b) allows for the simultaneous identification of blood vessel walls and structure and the characterization of blood flow. By using the same fluorophore for both of these markers, we were able to gain much information on vessel structure and blood flow dynamics from a single imaging channel, leaving maximal flexibility for the addition of other labels to identify and track multiple cell populations within these IVM imaging preparations. Although both imaging platforms provide a clear visualization of leukocyte behaviour at the single cell level, the image resolution is higher using multiphoton IVM. This increased resolution is in large part due to the absence of out-of-focus fluorescence originating from above or below the intended focal plane. In addition, multiphoton microscopy is able to image more deeply into the TME (up to 800 µm for multiphoton, compared to 150–200 µm of imaging depth possible when using confocal microscopy). As such, multiphoton imaging can support the capture of high-resolution images at a series of increasingly deeper focal planes. These individual focal planes can then be re-assembled to generate detailed 3D models of the TME (Video S2). Imaging tumors infected with an oncolytic virus further exemplifies the difference in resolution between resonant scanning confocal and multiphoton IVM (Figure 2c,d). Although resonant scanning confocal microscopy is able to facilitate the identification of individual tumor cells and can easily discern virally infected cells (Figure 2c), imaging the same tumour using the multiphoton platform reveals substantially more fine details. including numerous cellular processes (Figure 2d). Moreover, when imaging sequential focal planes of increasing depth through the tumor mass, resolution is rapidly lost from the images acquired using resonant scanning confocal microscopy, whereas image resolution is preserved when imaging is performed using multiphoton microscopy (Video S3).

The rapid imaging rates (>30 frames/s) achievable with a resonant scanning-based imaging platform make the acquisition of high-resolution mosaic images a practical approach to survey the overall tumor “landscape”. Through the use of a computer-driven microscope stage, it is possible to capture sequential, adjacent fields of view, completing a grid pattern that covers the entire surface of an exposed tissue or tumor. Once the images are acquired, individual image “tiles” can be aligned and merged into a single large image (Figure 2e). These high-resolution, low-magnification images accommodate the assessment of the overall tumor, identifying localized effects of immunotherapies such as oncolytic viral infection (green cells) within the overall tumor body (red cells). Importantly, each image tile represents a high-resolution, high-magnification image (Figure 2f), allowing for the determination of the behaviours and interactions on the single-cell level. Moreover, because of the speed of image acquisition, it is possible to acquire all image tiles in a matter of seconds. The imaging platform can be programmed to sequentially capture multiple tile scans. Once one mosaic image is captured, the microscope stage returns to its initial position and repeats the acquisition matrix. As a result, each image tile actually represents a video of several frames per second (Video S4), providing an opportunity for the analysis of cell trafficking and other dynamic processes within the overall mosaic image. In fact, since both the confocal and the multiphoton imaging platforms utilize the resonant scanner, high-resolution tile-scanned mosaic images can also be acquired using multiphoton technology (Figure 2g). Resonant scanning imaging rates are sufficiently fast to not only acquire videos for each tile in the mosaic but also enable the collection of a full stack of focal planes along the z-axis for each tile (image stack comprised of three z-planes, spaced 14 µm apart for a model of a 42-µm-thick tissue section). The end result is a 4D video, whereby a complete 3D structure can be tracked over time as a video (Video S5).

### 3.2. IVM of Leukocyte Behavior in Tumor Arterioles and Venules

**Figure 3:**
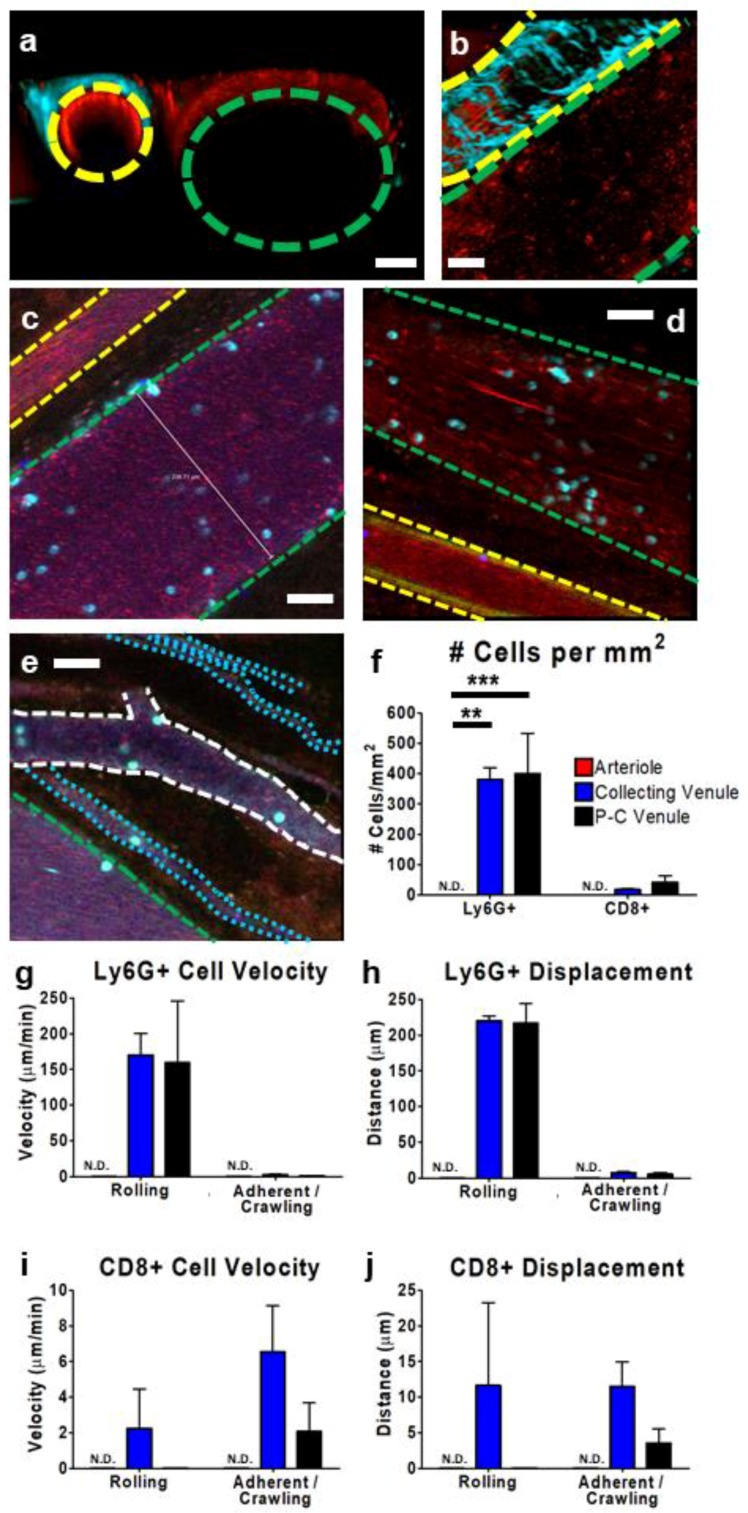
Characterization of tumor vasculature and leukocyte behavior within subcutaneous CT-26 tumor vessels. Representative IVM images (**a**–**e**) show the tumor vasculature. Using multiphoton imaging (**a**,**b**), differentiation between arterioles (yellow outline) and collecting venules (green outline) was facilitated by the observation of a collagen sheath (cyan-colored second-harmonic generation) surrounding the arteriole. Vasculature was highlighted by the presence of circulating platelets (red; PE-conjugated anti-CD49b), neutrophils (cyan; BV421-conjugated Ly6G), and CD8+ leukocytes (blue; eFluor 660-conjugated anti-CD8) and was imaged in either cross section (**a**) or transverse section (**b**). Using resonant scanning confocal microscopy (**c**–**e**), arterioles (yellow outline) were apparent as a result of increased autofluorescence (green) and collecting venules (green outline) were seen as parallel unbranching structures. In contrast, post-capillary venules (P-C venules) were observed as narrower, branching vessels (white outline), and tumor microcirculation/capillaries as very narrow (1–2 cell diameter) vessels (cyan outline) that followed a more convoluted path. Quantification of neutrophil (Ly6G+) and cytotoxic T cell (CD8+) interactions (cells present for ≥3 min) within arterioles (red), collecting venules (blue), and post-capillary venules (black) (**f**). Cell velocity (**g**,**i**) and displacement (**h**,**j**) of rolling and adherent and crawling neutrophils (**g**,**h**) and CD8+ T cells (**i**,**j**), as measured over a 10 min imaging period in a subcutaneous CT-26 tumour; *n* = 3 animals. Data displayed as the mean ± SEM. Total cell counts normalized for the area of each image occupied by a given vessel type. The white scale bar represents 50 µm. Statistical significance was determined using ANOVA; ** = *p* < 0.01; *** = *p* < 0.001; N.D. = not detected. Images in (**c**–**e**) were capture using resonant-scanning confocal microscopy, whereas images in (**a**,**b**) were captured using resonant scanning multiphoton microscopy.

The application of either multiphoton (Figure 3a,b) or confocal IVM (Figure 3c–e) to the TME enabled the identification and characterization of the tumor vasculature. Using multiphoton imaging, arteries and arterioles were clearly discernable because of the presence of collagen in the vessel wall (Figure 3a,b), a protein that naturally fluoresces as a result of second-harmonic generation (colored in cyan) [35,36,37,38]. In comparison, the collecting venules, ranging in diameter from 50 µm to >250 µm, appeared as thin-walled structures without collagen, had few branch points, and ran parallel to the arterioles. Using resonant scanning confocal imaging (Figure 3c,d), the arterioles were identified by their diameter, high flow rate, and the presence of a dim, auto-fluorescent signal detected at approximately 520 nm (clearly visible along the vessel walls in Figure 3d). Additionally, imaging revealed a much greater flow rate in the arterial circulation than in the venous circulation (Video S6). This imaging approach could also be used to characterize additional vessel structures, including the post-capillary venules (>50 µm, numerous branch points, no parallel arterioles) and the tumour microcirculation/capillaries (Figure 3e). Each of these various vascular structures plays a different role in gas and nutrient supply to the tumor, leukocyte infiltration, and delivery of therapeutics [36,37]. IVM allows us to directly observe and map how leukocytes interact with and behave within each of these vessel types.

In assessing cell recruitment, IVM can be used to directly quantify the number and type of leukocytes interacting with the vessel walls (Figure 3f). As previously reported in numerous other models of inflammation and immune challenge [39,40,41,42], leukocytes binding to or stably interacting with the vessel walls in the arterioles were not observed. In contrast, robust neutrophil recruitment and, to a much lesser extent, CD8+ cell recruitment was easily visualized in the collecting and post-capillary venules. Furthermore, IVM could provide information regarding the cellular behavior within these vessels. Leukocyte rolling (interacting with the vessel wall and displacing >1 cell diameter/5 min) could be measured within the different vessel types, collecting and post-capillary vessels (Figure 3g). Indeed, our preliminary analyses of these behaviors identified few crawling Ly6G+ neutrophils in the venules, with the non-rolling cells appearing fully adherent and not displacing from their initial position (Figure 3h). In contrast, CD8+ cells were observed to roll in post-capillary venules (Figure 3i) and crawl in both collecting and post-capillary venules (Figure 3j). It is important to note that the displacement and the distance travelled by a cell measure two fundamentally different parameters of cell movement. Displacement reports the linear distance between the starting location and end location of a given cell within a defined timeframe, whereas distance measures the length of the path the cell took to reach its destination. Often, within the vasculature, given the constraints of the vessel wall, displacement and distance travelled are very similar. This is not the case when studying cells within the tissue parenchyma (discussed further).

### 3.3. IVM of Leukocyte Behavior in Tumor Microcirculation/Capillaries

**Figure 4:**
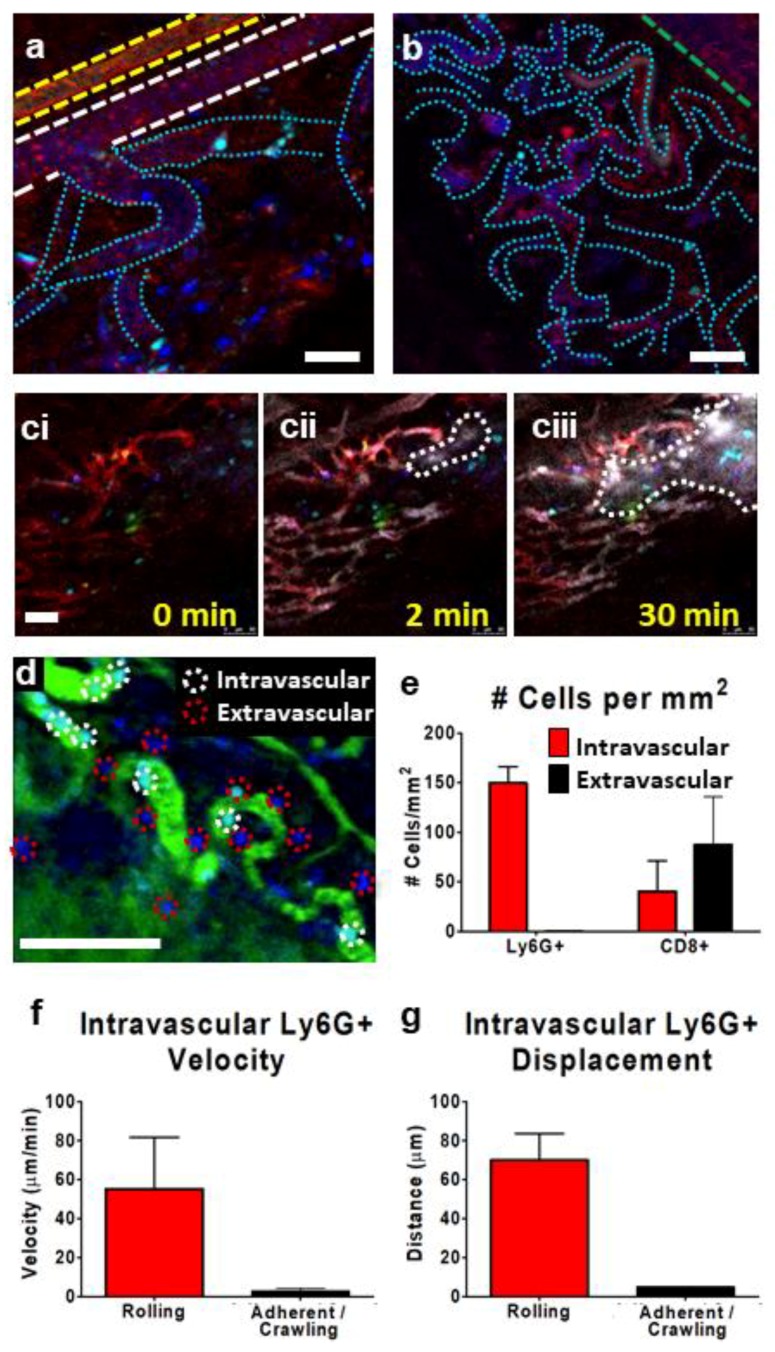
Characterization of subcutaneous CT-26 tumor microvasculature and leukocyte behavior. Representative IVM images (**a**–**c**) show the tumor microvasculature. The vasculature is highlighted by the presence of circulating platelets (red; PE-conjugated anti-CD49b), neutrophils (cyan; BV421-conjugated Ly6G), and CD8+ leukocytes (blue; eFluor 660-conjugated anti-CD8) (**a**,**b**). Using resonant scanning confocal microscopy, arterioles (yellow outline, **a**), veins (green outline, **b**) were seen as parallel unbranching structures. Venules were seen as narrower than veins and appeared as branching vessels (white outline, **a**), whereas capillaries/tumor microvasculature appeared as very narrow, convoluted vessels (cyan outline, **a**,**b**). Intravenous administration of fluorescent nanoparticles (Q-tracker; grey) identified areas of vascular leakage and accumulation of dye in the tissue interstitium (outlined in dotted white line) (**ci**–**ciii**). The introduction of FITC-conjugated albumin (green) allowed for an easy determination of intravascular (red circles) or extravascular (white circles) leukocytes (**d**). Quantification of intravascular (red) or extravascular (black) neutrophils (Ly6G+) and cytotoxic T cells (CD8+) (cells present for ≥3 min) within the tumor microvasculature (**e**). Velocity (**f**) and displacement (**g**) of rolling and adherent and crawling neutrophils as measured over a 10 min imaging period in a subcutaneous CT-26 tumor; *n* = 3 animals. Data displayed as the mean ± SEM. Total cell counts normalized for the area of each region of interest within each image (i.e., vessel vs. extravascular tissue). The white scale bar represents 50 µm. Images in (**a**–**d**) were captured using resonant scanning confocal microscopy.

In a similar fashion to arterioles and venules, IVM can be used to study leukocyte behavior within the tumor microcirculation/capillaries (Figure 4). The tumor microcirculation can assume a number of different physical appearances: gently meandering capillaries (Figure 4a) or a tumor-associated, highly convoluted and twisted microcirculation (Figure 4b). In both cases, the vessels possess the classic structural elements of capillaries: thin wall, narrow diameter, and slow blood flow; however, as the function in these vessels within the TME can differ dramatically from the classical capillaries within the skin, we hesitate to label the convoluted TME vasculature as capillaries and instead retain the more general term of microcirculation. The injection of a protein-coupled contrast agent or a nanocrystal-based contrast agent makes it possible to identify, track, and measure vascular permeability. Vascular permeability is of particular interest within tumors, as this phenomenon represents one potential mechanism whereby serum proteins and therapeutics are able to directly “leak” through gaps in the vascular endothelium, gaining direct access to the TME (Figure 4ci–ciii). In addition to visualizing the microcirculation by labelling the endothelium or by labelling circulating cells within the vessels, injection of a vascular contrast agent such as a fluorophore-conjugated protein (e.g., FITC-albumin) immediately and clearly defines the structure of the tumor vasculature (Figure 4d) and provides information regarding tissue perfusion. Using this approach to IVM of the tumor microvasculature, leukocyte location could be determined as either within the vessel lumen or in the extravascular parenchyma (Figure 4d,e). Additionally, just as in larger tumor vessels, IVM could be used to track the interactions between leukocytes and the microvascular endothelium, measuring cellular velocity (Figure 4f) and displacement (Figure 4g).

### 3.4. Tracking Leukocytes in the Tumor Interstitium by IVM

**Figure 5:**
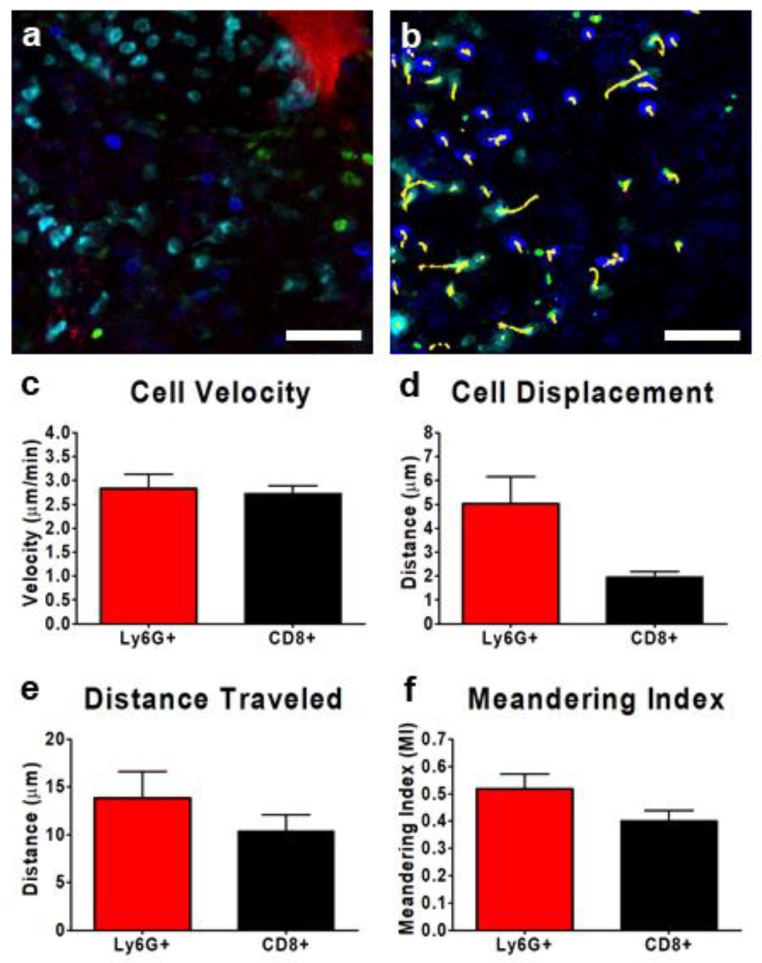
Characterization of interstitial leukocyte behavior within the TME of a subcutaneous CD-26 tumor. Representative resonant scanning confocal images of the tumor interstitium (**a**,**b**); tumor cells (red), neutrophils (cyan; BV421-conjugated Ly6G), and CD8+ leukocytes (blue; eFluor 660-conjugated anti-CD8) (**a**,**b**). Cell movement was tracked over a 10 min imaging window (yellow tracks) for at least three separate fields of view and quantified (**b**). Leukocyte velocity (**c**), displacement from initial starting point (**d**), distance travelled (**e**), and meandering index (**f**) were measured for each of the neutrophils (red) and CD8+ T cells (black); *n* = 3 animals. Between 8 and 50 Ly6G+ cells were traced in each field of view, whereas only 0–4 CD8+ cells were visualized per field of view. Data displayed as the mean ± SEM. The white scale bar represents 50 µm. Images in (**a**,**b**) were captured using resonant scanning confocal microscopy.

The use of IVM to track cells is not limited to the study of vascular-associated leukocytes. The application of IVM to the tumor interstitium enabled the visualization of both tumor resident cells, including leukocytes such as tumor-associated macrophages (TAMs), and recently recruited peripheral blood leukocytes that successfully egressed from the vasculature into the TME. This is illustrated in Figure 5 for Ly6G+ and CD8+ leukocytes. IVM could be used to track interstitial leukocyte behavior and identify cell–cell interactions, either with other leukocytes or with fluorescently-labeled tumor cells (Figure 5a). Using cell-tracking software, the movement of individual cells could be tracked over time, from seconds to hours, within the TME (Figure 5b, Video S7). Cellular movements could in turn be quantified, yielding information regarding leukocyte velocity (Figure 5c), cell displacement (Figure 5d), and the total distance travelled (Figure 5e). Importantly, as discussed earlier, displacement and distance travelled measure two fundamentally different aspects of leukocyte motility within a given tissue. Using both of these measurements, cell behavior such as the Meandering Index (MI) could be determined (Figure 5f). The MI is a description of how “direct” the movement of a cell is within a given tissue and is defined as the cell displacement divided by the total distance traveled. An MI of 1 indicates the cell moved in a direct, straight path from the starting point to the endpoint (such as a cell following a defined chemokine gradient), whereas a lower MI value suggests the cell took a “longer” route, meandering through the tissue, perhaps scanning for targets or following or crawling along specific structures within the TME (extracellular matrix, collagen, etc.).

## 4. Discussion

We presented here the capacity of IVM to study leukocyte recruitment to, and behavior within the TME in two syngeneic mouse models of cancer (subcutaneous colon carcinoma, intramuscular rhabdomyosarcoma). We showed how both resonant scanning confocal and resonant scanning multiphoton imaging platforms can be used to visualize tumor cells, tumor-associated vasculature, and various leukocyte populations. Although the concept and application of IVM has existed for more than a century, recent advances in imaging technologies have allowed for the widespread application of IVM to the study of the host immune response to cancer. Imaging platforms, such as spinning disk confocal microscopy, allow the user to visualize many dynamic processes in live animals. This early IVM platform allowed for rapid, multi-colored imaging of tissues, and, through the use of a CCD camera present in many spinning-disk systems, benefited from the ability to detect and capture weak fluorescent signals from tissues. Whereas imaging of some tissues has been relatively straightforward (liver, mesentery, muscle) [4,43,44], IVM of animal models of cancer has required the development of new technologies to address a number of unique technical limitations associated with tumors. IVM of tumors requires the ability to capture deep images (300–500 µm) within an often very dense tissue. Moreover, if IVM is used to study cells within tumor-associated vasculature, the imaging platform would require the ability to capture images at a very rapid rate (several frames per second).

The development of new, improved photodetectors and the advent of novel fluorophores has effectively addressed the issue of imaging depth. Specifically, the recent implementation of hybrid, HyD photodetectors [45] within a variety of imaging platforms has significantly increased imaging sensitivity, allowing for the capture of “dim” fluorescent signals originating deep within the tumor tissue. This technology, coupled with new, high quantum yield fluorophores, especially in the near-infrared spectral range, makes it possible to visualize structures at imaging depths (up to 800 µm) simply not possible with conventional light microscopy (typically less than 100 µm [46]) or basic fluorescent imaging.

With regards to imaging speed, both conventional laser scanning and multiphoton imaging platforms have limitations in the rate of image capture (1–3 frames/min), making these techniques suboptimal for imaging fast-moving targets such as circulating cells. The development of resonant scanning microscopy has, for the most part, overcome this limitation. The principle difference between laser scanning and resonant scanning microscopy is in how the various mirrors that are used to raster (scan back and forth) the laser across the sample are driven. In the past, these mirrors were controlled by small servo motors, whereas in resonant scanning microscopy the mirrors are controlled by a Piezo drive based on the ultrasonic vibration of crystals. This highly precise and extremely quick motor allows for imaging rates that are hundreds of times faster than those of conventional laser scanning confocal microscopy. This significant advancement now allows the tracking of cells within both the circulation and the TME.

**Figure 6:**
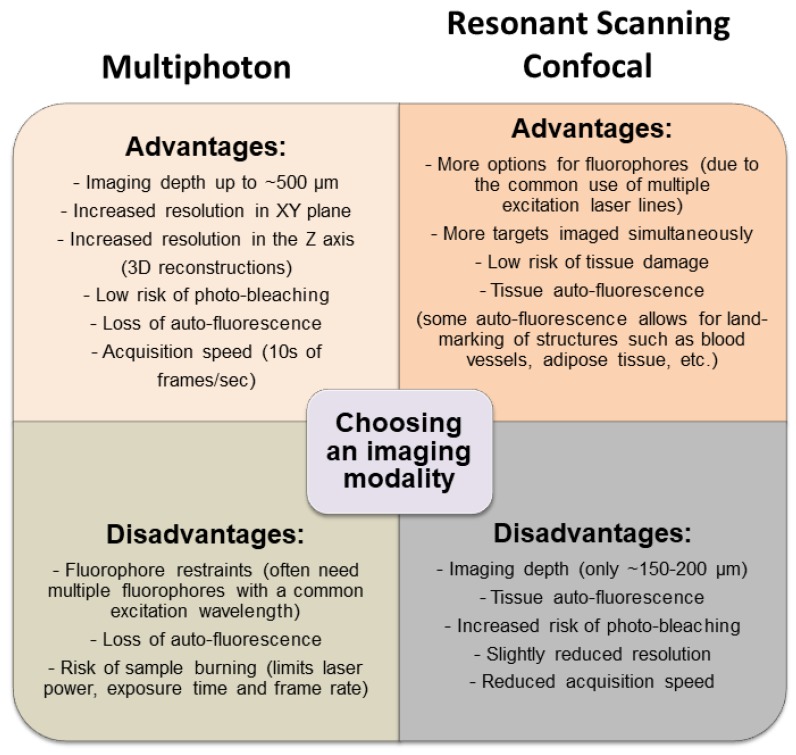
Comparing resonant scanning confocal versus multiphoton IVM imaging for studying cell recruitment and behavior in the tumor microenvironment (TME). The advantages and disadvantages of each imaging modality are listed to provide a basis for choosing which technique would best suit a specific experiment.

Although both resonant scanning confocal microscopy and multiphoton microscopy are able to visualize leukocyte behavior within the TME, each has its own advantages and disadvantages (Figure 6). Whereas confocal microscopy is best equipped to simultaneously visualize numerous cell markers (due to the ability to add excitation laser lines at a reasonable cost), this imaging modality is not optimal for imaging deep structures (beyond 200 µm). Additionally, resonant scanning confocal causes less thermal damage to tissues and provides greater flexibility with respect to the control of tissue auto-florescence but sacrifices some image resolution in the process. In contrast, whereas multiphoton imaging is better for visualizing deep structures (400 to 800 µm) and provides improved image resolution, it sacrifices flexibility in fluorophore selection and ability to image more than three or four markers within a single tissue preparation (due to the cost of adding additional laser lines), although generally cost-prohibitive, multiphoton platforms with two and even three excitation laser lines are becoming more common, greatly enhancing the ability to simultaneously image multiple markers. Additionally, multiphoton imaging is less forgiving with regards to tissue movement, a factor that must be considered when imaging live animals. The choice of a specific imaging platform for a given experimental question depends largely on the balance of these “advantages” and “disadvantages”, so that one platform may be optimal for one tumor model but perhaps not all models.

In the current study, we demonstrated that IVM is a robust platform to visualize and characterize tumor structure, vascular composition, and leukocyte recruitment to the TME. It is important to note that the ability to determine statistical differences between various groups of animals using IVM is largely dependent on the specific question being asked. Specifically, with respect to power calculations and the number of animals needed to prove the existence of a statistical difference (or absence of a difference), it is directly related to the parameters being measured. When one is simply enumerating cells, typically 5–6 animals per group are sufficient to determine if a statistical difference exists or not. When measuring parameters with a greater degree of variability (deposition of extracellular matrix), rare events (dynamic cell–cell interactions), or phenomena that are not evenly distributed throughout the tissue (dynamics of vascular leaks), additional animals are often required (12–15). Importantly, although it is a powerful tool, IVM does not necessarily capture a picture of an entire tissue or organ. If, for example, a tumor is small, IVM will allow for the assessment and characterization of most of the tissue; however, if a tumor is large, IVM will only allow for an assessment of a fraction of the whole tumor and, as such, other techniques may be required, partnered with IVM, to provide an overall assessment of the target tissue.

In summary, IVM allows for the observation, characterization, and quantification of dynamic processes within the TME. These processes include leukocyte–endothelial cell, leukocyte–tumor cell, and leukocyte–leukocyte interactions within the tumor vasculature and interstitium. In this work, we have highlighted how IVM can be used to characterize tumor structure and tumor vasculature and to track leukocyte recruitment and behavior within the TME. This ability to “watch” the immune response will provide key insights into the effect of various anti-cancer treatments, enabling the direct assessment, for example, of the ability of a given therapeutic to enhance leukocyte recruitment or to stabilize lymphocyte–cancer cell interactions. Moreover, we can directly determine where, within the microvascular network, leukocyte adhesion occurs and where these cells are able to exit the vasculature and enter the tissue interstitium. These IVM approaches will play an integral role in our evolving understanding of the TME and will allow us to better design and optimize treatments to ensure efficient leukocyte recruitment to, and access into, tumors with the overall goal of enhancing tumour clearance.

## Supplementary Materials

The following are available online at http://www.mdpi.com/2073-4409/7/7/69/s1, Video S1: Resonant-Scanning Confocal and Multiphoton Imaging of Tumor Vasculature. Video S2: 3D-Reconstruction of Image Z-stack From Multiphoton Imaging of Tumor Vasculature. Video S3: Resonant Scanning Confocal and Multiphoton Imaging of Focal Planes of Increasing Depth in a Virally Infected Tumor. Video S4: Video extracted from a Single Tile of a Mosaic Image Obtained by Resonant Scanning Confocal Imaging of Tumor Vasculature. Video S5: 4D-Reconstruction of Tile Scan Z-stack From Multiphoton Imaging of Tumor Vasculature. Video S6: Intravital Characterization and Imaging of Tumor Vasculature. Video S7: 3 h IVM of Tumor Vasculature.

## References

[1] Cohnheim J. (1872). Untersuchungen über die Embolischen Process.

[2] Waller A. (1846). Microscopic observations on the perforation of the capillaries by the corpuscles of the blood, and on the origin of mucus and pus-globules. Lond. Edinb. Dublin Philos. Mag..

[3] Hall M. (1831). A Critical and Experimental Essay on the Circulation of the Blood.

[4] Johnston B., Burns A.R., Suematsu M., Issekutz T.B., Woodman R.C., Kubes P. (1999). Chronic inflammation upregulates chemokine receptors and induces neutrophil migration to monocyte chemoattractant protein-1. J. Clin. Investig..

[5] Ho M., Hickey M.J., Murray A.G., Andonegui G., Kubes P. (2000). Visualization of Plasmodium falciparum-endothelium interactions in human microvasculature: Mimicry of leukocyte recruitment. J. Exp. Med..

[6] Allen C.D., Okada T., Tang H.L., Cyster J.G. (2007). Imaging of germinal center selection events during affinity maturation. Science.

[7] Zeng Z., Surewaard B.G., Wong C.H., Geoghegan J.A., Jenne C.N., Kubes P. (2016). CRIg Functions as a Macrophage Pattern Recognition Receptor to Directly Bind and Capture Blood-Borne Gram-Positive Bacteria. Cell Host Microbe.

[8] Wang J., Hossain M., Thanabalasuriar A., Gunzer M., Meininger C., Kubes P. (2017). Visualizing the function and fate of neutrophils in sterile injury and repair. Science.

[9] Stremmel C., Schuchert R., Wagner F., Thaler R., Weinberger T., Pick R., Mass E., Ishikawa-Ankerhold H.C., Margraf A., Hutter S. (2018). Yolk sac macrophage progenitors traffic to the embryo during defined stages of development. Nat. Commun..

[10] Perez-Camps M., Tian J., Chng S.C., Sem K.P., Sudhaharan T., Teh C., Wachsmuth M., Korzh V., Ahmed S., Reversade B. (2016). Quantitative imaging reveals real-time Pou5f3-Nanog complexes driving dorsoventral mesendoderm patterning in zebrafish. eLife.

[11] Guidotti L.G., Inverso D., Sironi L., Di L.P., Fioravanti J., Ganzer L., Fiocchi A., Vacca M., Aiolfi R., Sammicheli S. (2015). Immunosurveillance of the liver by intravascular effector CD8(+) T cells. Cell.

[12] Jenne C.N., Wong C.H., Zemp F.J., McDonald B., Rahman M.M., Forsyth P.A., McFadden G., Kubes P. (2013). Neutrophils recruited to sites of infection protect from virus challenge by releasing neutrophil extracellular traps. Cell Host Microbe.

[13] McDonald B., Davis R.P., Kim S.J., Tse M., Esmon C.T., Kolaczkowska E., Jenne C.N. (2017). Platelets and neutrophil extracellular traps collaborate to promote intravascular coagulation during sepsis in mice. Blood.

[14] Christoffersson G., Vagesjo E., Vandooren J., Liden M., Massena S., Reinert R.B., Brissova M., Powers A.C., Opdenakker G., Phillipson M. (2012). VEGF-A recruits a proangiogenic MMP-9-delivering neutrophil subset that induces angiogenesis in transplanted hypoxic tissue. Blood.

[15] Woodfin A., Voisin M.B., Beyrau M., Colom B., Caille D., Diapouli F.M., Nash G.B., Chavakis T., Albelda S.M., Rainger G.E. (2011). The junctional adhesion molecule JAM-C regulates polarized transendothelial migration of neutrophils in vivo. Nat. Immunol..

[16] Dal-Secco D., Wang J., Zeng Z., Kolaczkowska E., Wong C.H., Petri B., Ransohoff R.M., Charo I.F., Jenne C.N., Kubes P. (2015). A dynamic spectrum of monocytes arising from the in situ reprogramming of CCR2+ monocytes at a site of sterile injury. J. Exp. Med..

[17] McDonald B., Pittman K., Menezes G.B., Hirota S.A., Slaba I., Waterhouse C.C., Beck P.L., Muruve D.A., Kubes P. (2010). Intravascular danger signals guide neutrophils to sites of sterile inflammation. Science.

[18] Phan T.G., Green J.A., Gray E.E., Xu Y., Cyster J.G. (2009). Immune complex relay by subcapsular sinus macrophages and noncognate B cells drives antibody affinity maturation. Nat. Immunol..

[19] Lammermann T., Afonso P.V., Angermann B.R., Wang J.M., Kastenmuller W., Parent C.A., Germain R.N. (2013). Neutrophil swarms require LTB4 and integrins at sites of cell death in vivo. Nature.

[20] Martinez A.F., McCachren S.S., Lee M., Murphy H.A., Zhu C., Crouch B.T., Martin H.L., Erkanli A., Rajaram N., Ashcraft K.A. (2018). Metaboloptics: Visualization of the tumor functional landscape via metabolic and vascular imaging. Sci. Rep..

[21] Ikeda W., Sasai K., Akagi T. (2018). Imaging Window Device for Subcutaneous Implantation Tumor. Methods Mol. Biol..

[22] Seynhaeve A.L., Ten Hagen T.L. (2016). High-Resolution Intravital Microscopy of Tumor Angiogenesis. Methods Mol. Biol..

[23] Benjamin D.C., Hynes R.O. (2017). Intravital imaging of metastasis in adult Zebrafish. BMC Cancer.

[24] Babes L., Kubes P. (2016). Visualizing the Tumor Microenvironment of Liver Metastasis by Spinning Disk Confocal Microscopy. Methods Mol. Biol..

[25] Weber M.R., Zuka M., Lorger M., Tschan M., Torbett B.E., Zijlstra A., Quigley J.P., Staflin K., Eliceiri B.P., Krueger J.S. (2016). Activated tumor cell integrin alphavbeta3 cooperates with platelets to promote extravasation and metastasis from the blood stream. Thromb. Res..

[26] Madsen D.H., Jurgensen H.J., Siersbaek M.S., Kuczek D.E., Grey C.L., Liu S., Behrendt N., Grontved L., Weigert R., Bugge T.H. (2017). Tumor-Associated Macrophages Derived from Circulating Inflammatory Monocytes Degrade Collagen through Cellular Uptake. Cell Rep..

[27] Belli C., Trapani D., Viale G., D’Amico P., Duso B.A., Della V.P., Orsi F., Curigliano G. (2018). Targeting the microenvironment in solid tumors. Cancer Treat. Rev..

[28] Binnewies M., Roberts E.W., Kersten K., Chan V., Fearon D.F., Merad M., Coussens L.M., Gabrilovich D.I., Ostrand-Rosenberg S., Hedrick C.C. (2018). Understanding the tumor immune microenvironment (TIME) for effective therapy. Nat. Med..

[29] Dzhandzhugazyan K.N., Guldberg P., Kirkin A.F. (2018). Adoptive T cell cancer therapy. Nat. Mater..

[30] Mahoney D.J., Stojdl D.F., Laird G. (2014). Virus therapy for cancer. Sci. Am..

[31] Kim D.S., Dastidar H., Zhang C., Zemp F.J., Lau K., Ernst M., Rakic A., Sikdar S., Rajwani J., Naumenko V. (2017). Smac mimetics and oncolytic viruses synergize in driving anticancer T-cell responses through complementary mechanisms. Nat. Commun..

[32] Naumenko V., Jenne C., Mahoney D.J. (2016). Intravital Microscopy for Imaging the Tumor Microenvironment in Live Mice. Methods Mol. Biol..

[33] Schindelin J., Rganda-Carreras I., Frise E., Kaynig V., Longair M., Pietzsch T., Preibisch S., Rueden C., Saalfeld S., Schmid B. (2012). Fiji: An open-source platform for biological-image analysis. Nat. Methods.

[34] Parslow A., Cardona A., Bryson-Richardson R.J. (2014). Sample drift correction following 4D confocal time-lapse imaging. J. Vis. Exp..

[35] Gailhouste L., Le G.Y., Odin C., Guyader D., Turlin B., Ezan F., Desille Y., Guilbert T., Bessard A., Fremin C. (2010). Fibrillar collagen scoring by second harmonic microscopy: A new tool in the assessment of liver fibrosis. J. Hepatol..

[36] Braverman I.M. (2000). The cutaneous microcirculation. J. Investig. Dermatol. Symp. Proc..

[37] Pober J.S., Sessa W.C. (2014). Inflammation and the blood microvascular system. Cold Spring Harb. Perspect. Biol..

[38] Lemaster K.A., Farid Z., Brock R.W., Shrader C.D., Goldman D., Jackson D.N., Frisbee J.C. (2017). Altered post-capillary and collecting venular reactivity in skeletal muscle with metabolic syndrome. J. Physiol..

[39] Kubes P., Kanwar S. (1994). Histamine induces leukocyte rolling in post-capillary venules. A P-selectin-mediated event. J. Immunol..

[40] Jung U., Ley K. (1999). Mice lacking two or all three selectins demonstrate overlapping and distinct functions for each selectin. J. Immunol..

[41] Von Andrian U.H., Mackay C.R. (2000). T-cell function and migration. Two sides of the same coin. N. Engl. J. Med..

[42] Al F.H., Kang J.H., Hwang S.R., Sung S., Alam M.M., Sa K.H., Nam E.J., Byun Y.R., Kang Y.M. (2017). Stepwise inhibition of T cell recruitment at post-capillary venules by orally active desulfated heparins in inflammatory arthritis. PLoS ONE.

[43] Horie Y., Wolf R., Chervenak R.P., Jennings S.R., Granger D.N. (1999). T-lymphocytes contribute to hepatic leukostasis and hypoxic stress induced by gut ischemia-reperfusion. Microcirculation.

[44] Petri B., Broermann A., Li H., Khandoga A.G., Zarbock A., Krombach F., Goerge T., Schneider S.W., Jones C., Nieswandt B. (2010). Von Willebrand factor promotes leukocyte extravasation. Blood.

[45] Borlinghaus R.T. (2015). Sensors and Measuring Techniques in Confocal Microscopy.

[46] Kim A.H., Suleiman H., Shaw A.S. (2016). New approaches in renal microscopy: Volumetric imaging and superresolution microscopy. Curr. Opin. Nephrol. Hypertens..

