# Supplementary material for: Tracking Cell Recruitment and Behavior within the Tumor Microenvironment Using Advanced Intravital Imaging Approaches
Source: Cells. 2018 Jul 3;7(7):69. doi: 10.3390/cells7070069 (PMC6071013; doi:10.3390/cells7070069)
Supplement: Supplementary file 1 [file cells-07-00069-s001.zip › Figure 1.pdf]

Figure 1

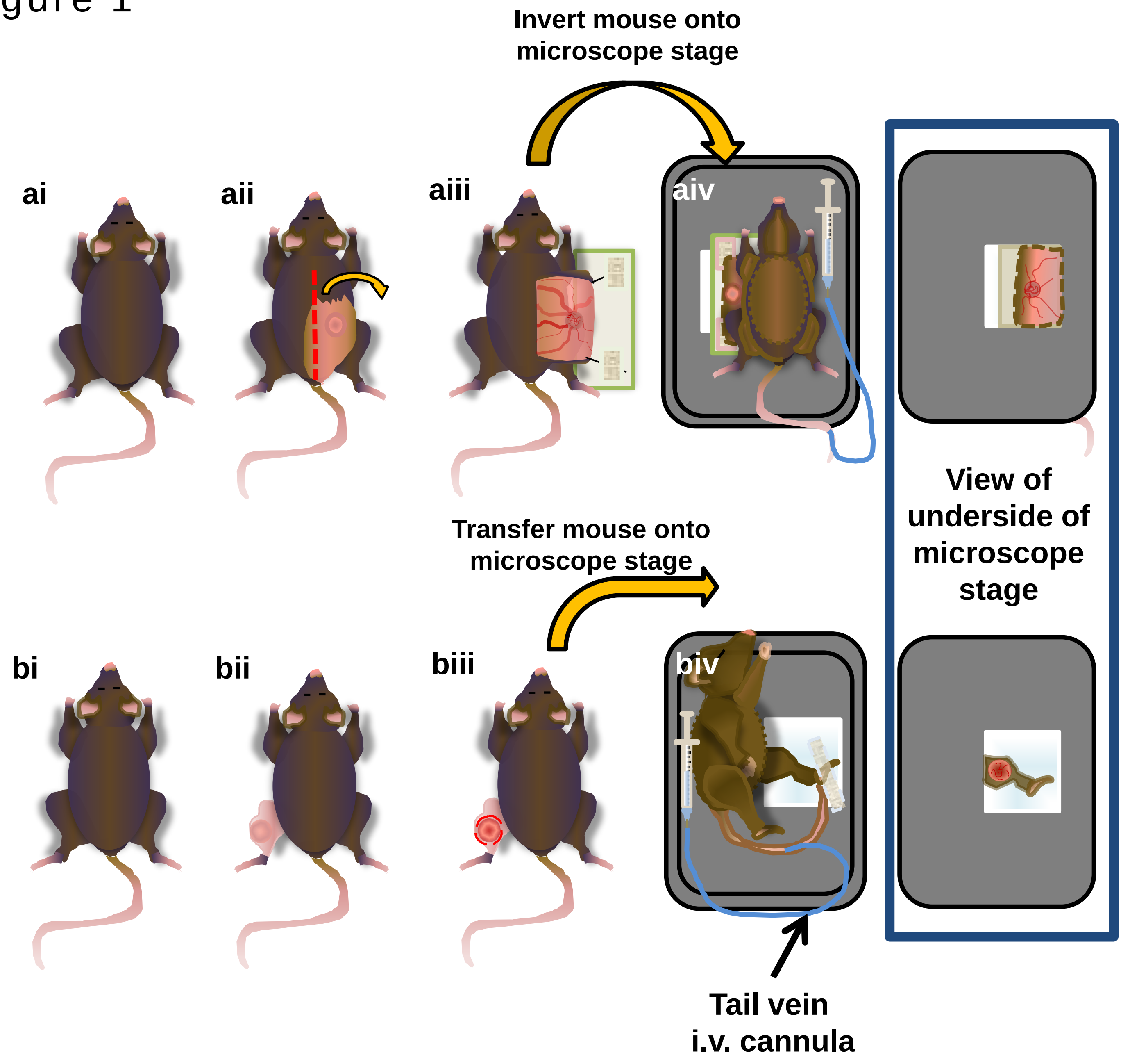

Figure 1. Surgical preparation of subcutaneous and intramuscular tumours for IVM imaging. Mice are either injected with tumour cells subcutaneously on their flank **(a)** or intramuscularly in the gastrocnemius of the leg **(b)**. After approximately 10 days, tumours are exposed **(aii, bii)** and tissue movment is surgically stabilized **(aiii, biii)** and the mouse is inverted and placed onto a heated (37° C) microscope stage **(aiv, biv)**. An i.v. cannula is inserted into the tail vein to provide anesthetic when necessary throughout the imaging procedure.
