# Supplementary material for: Tracking Cell Recruitment and Behavior within the Tumor Microenvironment Using Advanced Intravital Imaging Approaches
Source: Cells. 2018 Jul 3;7(7):69. doi: 10.3390/cells7070069 (PMC6071013; doi:10.3390/cells7070069)
Supplement: Supplementary file 1 [file cells-07-00069-s001.zip › Figure 2.pdf]

Figure 2

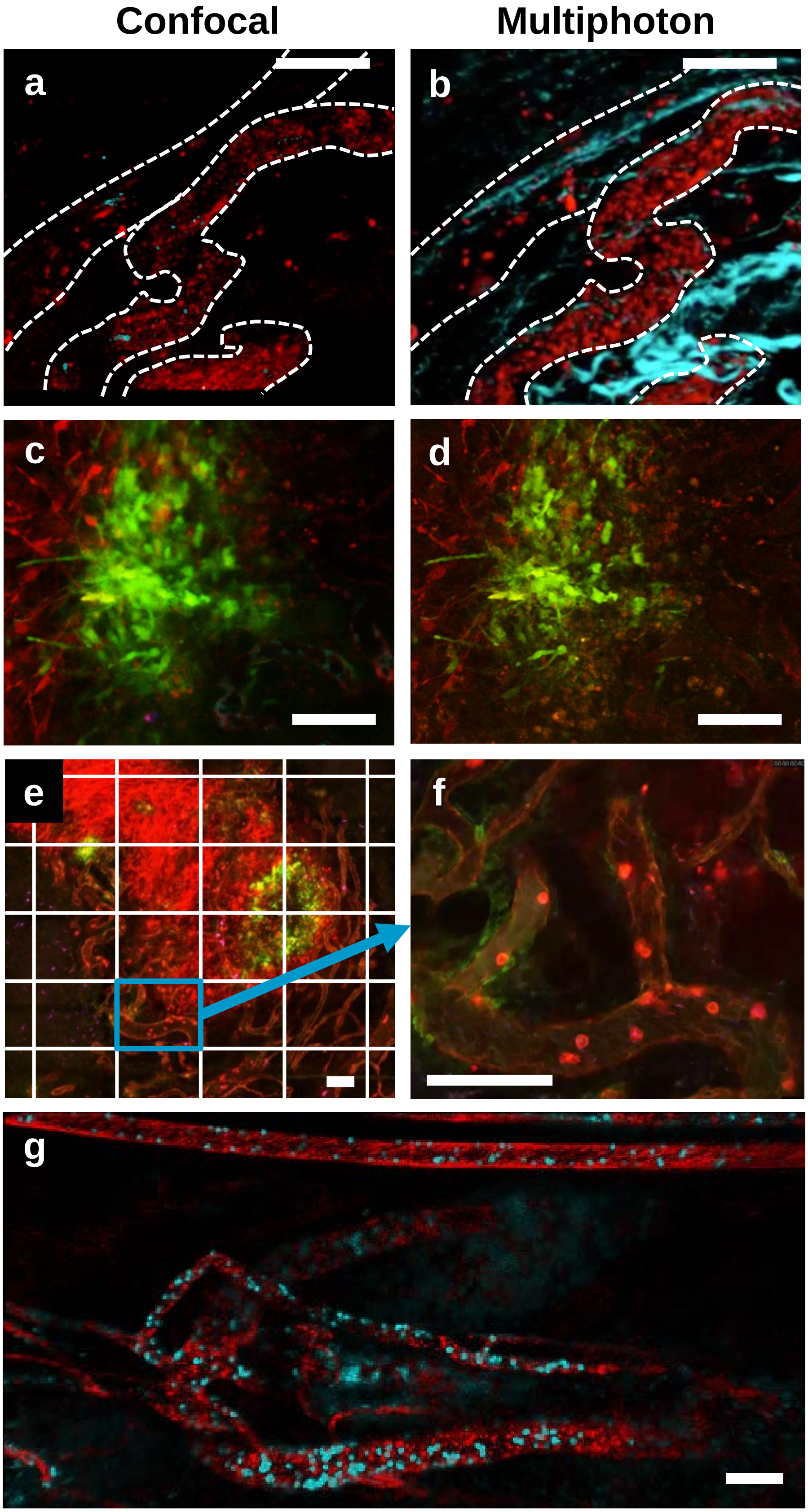

Figure 2. IVM of tumour vasculature acquired with resonant scanning confocal or multiphoton imaging modalities. Representative images of a single field of view showing a subcutaneous CT-26 tumour vasculature (red; PE-conjugated anti-CD31 and PE-conjugated anti-CD49b, blood vessels are denoted by dotted white outline) captured with either confocal **(a)** or multiphoton **(b)** imaging (collagen appears blue as a result of second harmonic generation). In the confocal image **(a)**, some CD8+ cells (blue; eFluor 660-conjugated anti-CD8α) are visible; however, these cells are not seen in the multiphoton image **(b)** due to a lack of fluorophore stimulation by the single multiphoton excitation wavelength used in this imaging. Confocal **(c)** and multiphoton **(d)** imaging of a subcutaneous CT-26 tumour (red) infected with VSV that is transgenic for GFP ($VSV^{\Delta M51\text{-}GFP}$; green). A stitched, tile scan image **(e)** of an entire subcutaneous CT-26 tumour (bright red) demonstrating localized VSV infection (green) was captured using resonant-scanning confocal imaging. Note each tiled image (outlined by the white grid) is captured as a high resolution video **(f)** allowing for later analysis at the cellular level (neutrophils in bright red, endothelium in dim red, i.v. delivered eFluor 660-labelled $VSV^{\Delta M51\text{-}GFP}$ in blue). High resolution 4D movies of stitched images **(g)** of the tumour vasculature of a rhabdomyosarcoma within the gastrocnemius muscle (red; PE-conjugated anti-CD31 and PE-conjugated anti-CD49b), containing neutrophils (cyan; BV421-conjugated anti-Ly6G) captured using multiphoton microscopy. White scale bar represents 100 µm. Images in **(a, c, e, f)** were capture using resonant-scanning confocal microscopy whereas images in **(b, d, g)** were captured using resonant-scanning multiphoton microscopy.
