# Supplementary material for: Tracking Cell Recruitment and Behavior within the Tumor Microenvironment Using Advanced Intravital Imaging Approaches
Source: Cells. 2018 Jul 3;7(7):69. doi: 10.3390/cells7070069 (PMC6071013; doi:10.3390/cells7070069)
Supplement: Supplementary file 1 [file cells-07-00069-s001.zip › Figure 3.pdf]

Figure 3

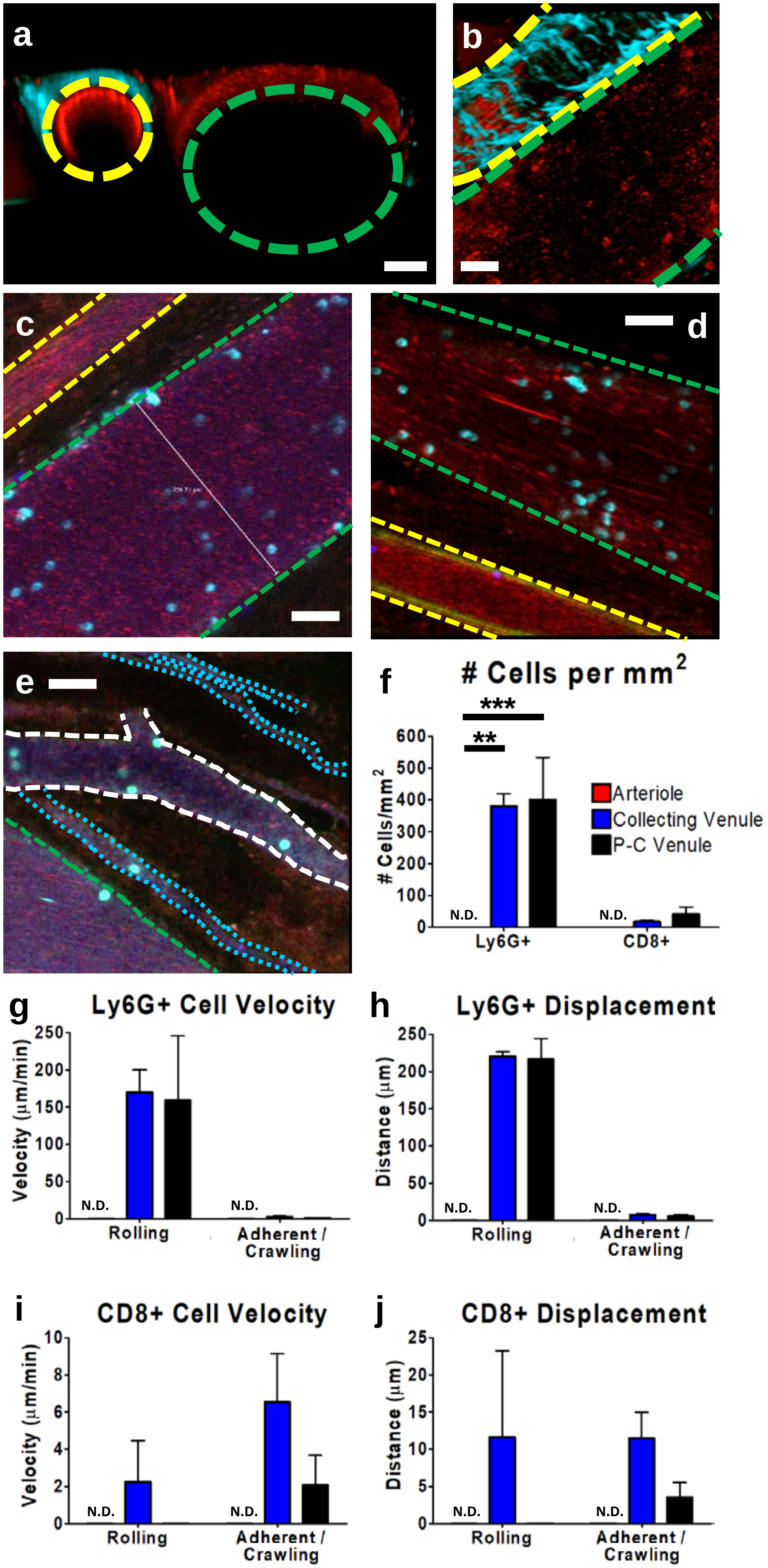

a
b
c
d
e
f
Cells per mm2
***
**
600
500
400
300
200
100
0
Cells/mm2
N.D.
N.D.
Ly6G+
CD8+
Arteriole
Collecting Venule
P-C Venule
g
Ly6G+ Cell Velocity
Velocity (μm/min)
250
200
150
100
50
0
N.D.
N.D.
Rolling
Adherent / Crawling
h
Ly6G+ Displacement
Distance (μm)
250
200
150
100
50
0
N.D.
N.D.
Rolling
Adherent / Crawling
i
CD8+ Cell Velocity
Velocity (μm/min)
10
8
6
4
2
0
N.D.
N.D.
Rolling
Adherent / Crawling
j
CD8+ Displacement
Distance (μm)
25
20
15
10
5
0
N.D.
N.D.
Rolling
Adherent / Crawling

Figure 3. Characterization of tumour vasculature and leukocyte behaviour within subcutaneous CT-26 tumour vessels. Representative IVM images **(a-e)** show the tumour vasculature. Using multiphoton imaging **(a, b)**, differentiation between arterioles (yellow outline) and collecting venules (green outline) is facilitated by the observation of a collagen sheath (cyan coloured second harmonic generation) surrounding the arteriole. Vasculature is highlighted by the presence of circulating platelets (red; PE-conjugated anti-CD49b), neutrophils (cyan; BV421-conjugated Ly6G) and CD8+ leukocytes (blue; eFluor 660-conjugated anti-CD8) and is imaged in either cross-section **(a)** or in transverse-section **(b)**. Using resonant-scanning confocal microscopy **(c-e)**, arterioles (yellow outline) are apparent due to increased autofluorescence (green) and collecting venules (green outline) are seen as parallel unbranching structures. In contrast, post-capillary venules (P-C venules) are observed as narrower, branching vessels (white outline) and tumour microcirculation / capillaries as very narrow (1-2 cell diameter) vessels (cyan outline) that follow a more convoluted path. Quantification of neutrophil (Ly6G+) and cytotoxic T cell (CD8+) interactions (cells present for ≥3 min) within arterioles (red), collecting venules (blue) and post-capillary venules (black) **(f)**. Cell velocity **(g, i)** and displacement **(h, j)** of rolling and adherent/crawling neutrophils **(g, h)** and CD8+ T cells **(i, j)** as measured over a 10 min imaging period in a subcutaneous CT-26 tumour. n = 3 animals. Data displayed as the mean ±SEM. Total cell counts normalized for the area of each image occupied by a given vessel type. White scale bar represents 50 µm. Statistical significance was determined using ANOVA. ** = $p < 0.01$; *** = $p < 0.001$; N.D. = not detected. Images in **(c, d, e)** were capture using resonant-scanning confocal microscopy whereas images in **(a, b)** were captured using resonant-scanning multiphoton microscopy.
