# Supplementary material for: Tracking Cell Recruitment and Behavior within the Tumor Microenvironment Using Advanced Intravital Imaging Approaches
Source: Cells. 2018 Jul 3;7(7):69. doi: 10.3390/cells7070069 (PMC6071013; doi:10.3390/cells7070069)
Supplement: Supplementary file 1 [file cells-07-00069-s001.zip › Figure 4.pdf]

Figure 4

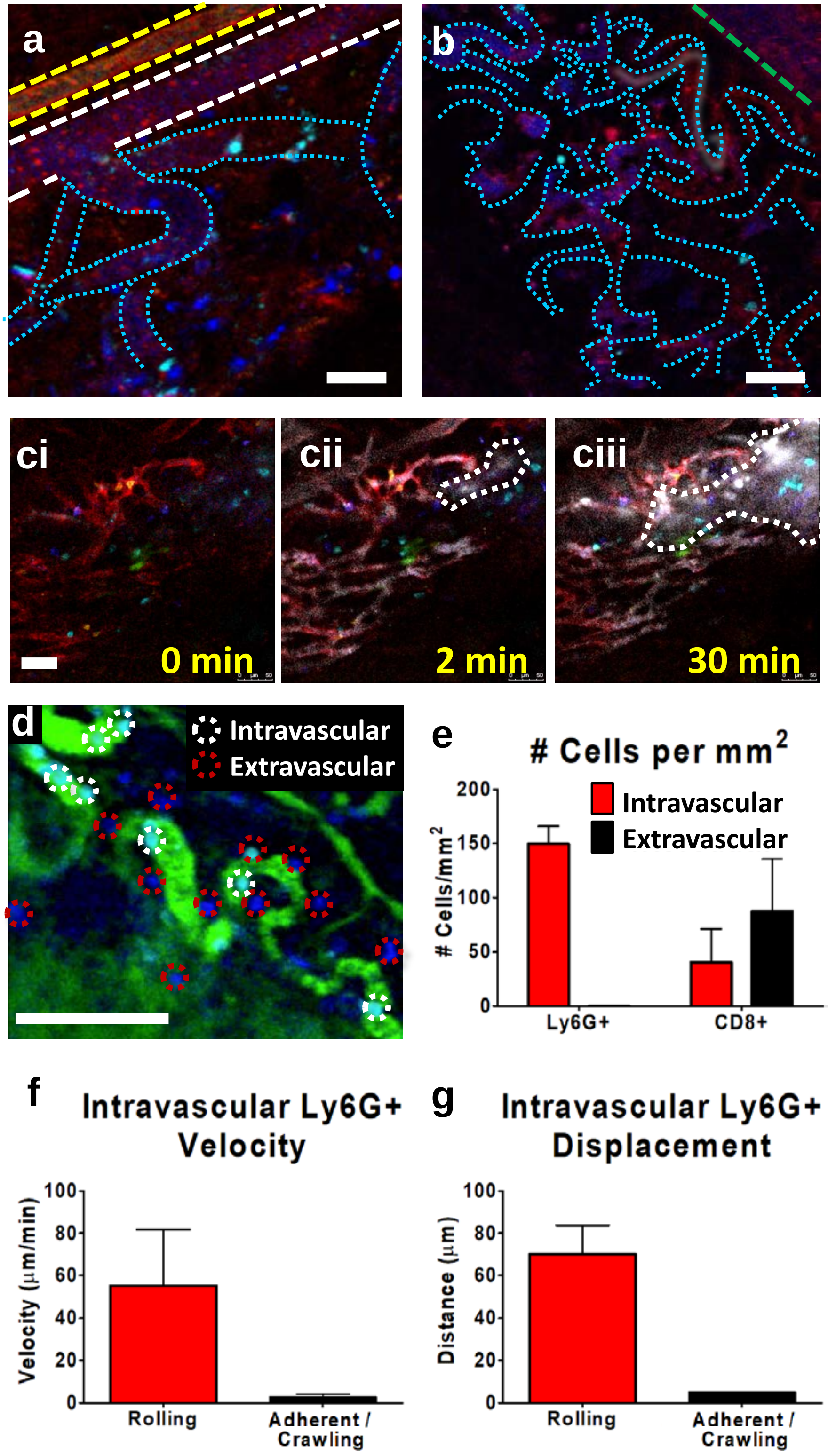

a
b
ci
0 min
cii
2 min
ciii
30 min
d
Intravascular
Extravascular
e
Cells per mm^2
Intravascular
Extravascular
Cells/mm^2
200
150
100
50
0
Ly6G+
CD8+
f
Intravascular Ly6G+
Velocity
Velocity (μm/min)
100
80
60
40
20
0
Rolling
Adherent / Crawling
g
Intravascular Ly6G+
Displacement
Distance (μm)
100
80
60
40
20
0
Rolling
Adherent / Crawling

Figure 4. Characterization of subcutaneous CT-26 tumour microvasculature and leukocyte behaviour. Representative IVM images **(a-c)** show the tumour microvasculature. Vasculature is highlighted by the presence of circulating platelets (red; PE-conjugated anti-CD49b), neutrophils (cyan; BV421-conjugated Ly6G) and CD8+ leukocytes (blue; eFluor 660-conjugated anti-CD8) **(a, b)**. Using resonant-scanning confocal microscopy, arterioles (yellow outline, a), veins (green outline, **b**) are seen as parallel unbranching structures. Venules are seen as narrower than veins and appear as branching vessels (white outline, **a**) whereas capillaries/tumour microvasculature appear as very narrow, convoluted vessels (cyan outline, **a-b**). Intravenous administration of fluorescent nanoparticles (Q-tracker; grey) identifies areas of vascular leakage and accumulation of dye in the tissue interstitium (outlined in dotted white line) **(ci-ciii)**. Introduction of FITC-conjugated albumin (green) allows for easy determination of intravascular (red circles) or extravascular (white circles) leukocytes **(d)**. Quantification of intravascular (red) or extravascular (black) neutrophils (Ly6G+) and cytotoxic T cells (CD8+) (cells present for ≥3 min) within the tumour microvasculature **(e)**. Velocity **(f)** and displacement **(g)** of rolling and adherent/crawling neutrophils as measured over a 10 min imaging period in a subcutaneous CT-26 tumour. n = 3 animals. Data displayed as the mean ±SEM. Total cell counts normalized for the area of each region of interst within in each image (i.e. vessel vs. extravasscular tissue). White scale bar represents 50 µm. Images in **(a, b, c, d)** were capture using resonant-scanning confocal microscopy.
