# Supplementary material for: Tracking Cell Recruitment and Behavior within the Tumor Microenvironment Using Advanced Intravital Imaging Approaches
Source: Cells. 2018 Jul 3;7(7):69. doi: 10.3390/cells7070069 (PMC6071013; doi:10.3390/cells7070069)
Supplement: Supplementary file 1 [file cells-07-00069-s001.zip › Figure 5.pdf]

Figure 5

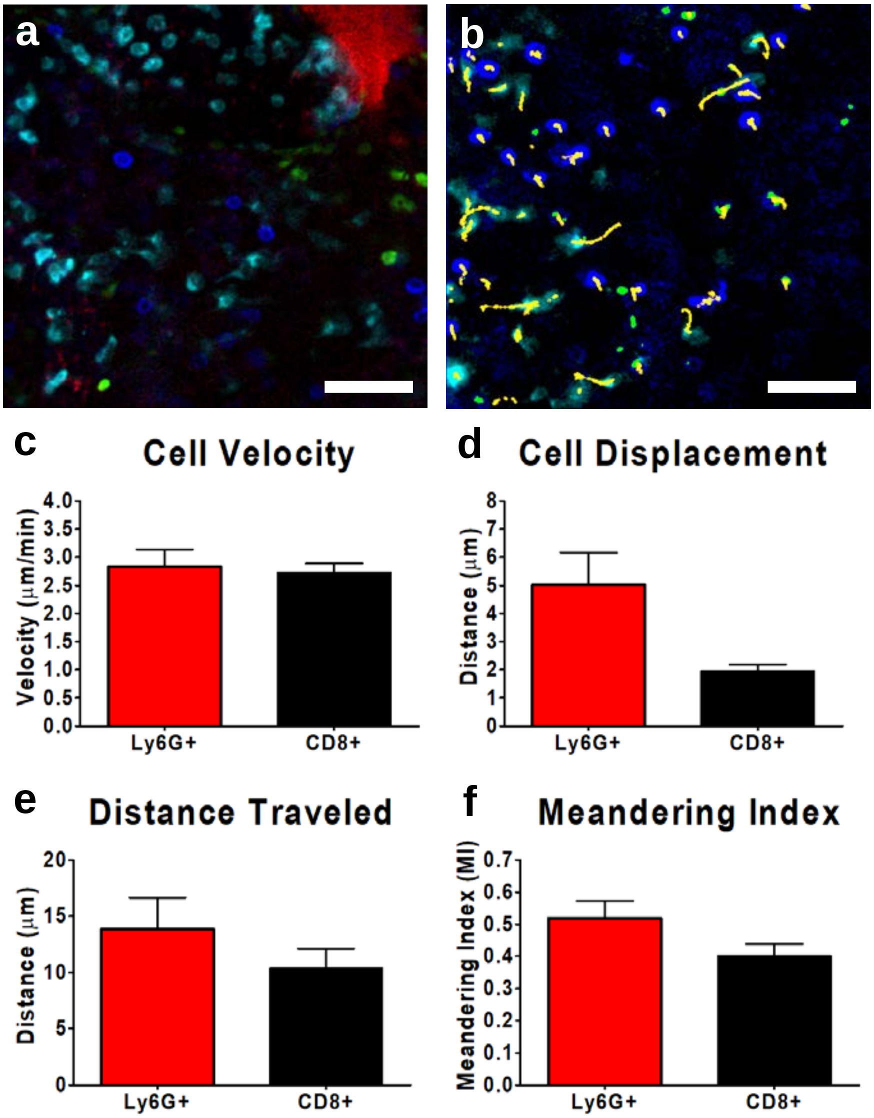

Figure 5. Characterization of interstitial leukocyte behaviour within the TME of a subcutaneous CD-26 tumour. Representative resonant-scanning confocal images of the tumour interstitium **(a-b)**; tumour cells (red), neutrophils (cyan; BV421-conjugated Ly6G) and CD8+ leukocytes (blue; eFluor 660-conjugated anti-CD8) **(a, b)**. Cell movement was tracked over a 10 min imaging window (yellow tracks) for at least 3 separate fields of view and quantified **(b)**. Leukocyte velocity **(c)**, displacement from initial starting point **(d)**, distance travelled **(e)**, and meandering index **(f)** were measured for each of the neutrophils (red) and CD8+ T cells (black). n = 3 animals. Between 8-50 Ly6G+ cells were tracted in each field of view where as only 0-4 CD8+ cells were visulaized per field of view. Data displayed as the mean ±SEM. White scale bar represents 50 µm. Images in **(a, b)** were capture using resonant-scanning confocal microscopy.
