# Supplementary material for: Tracking Cell Recruitment and Behavior within the Tumor Microenvironment Using Advanced Intravital Imaging Approaches
Source: Cells. 2018 Jul 3;7(7):69. doi: 10.3390/cells7070069 (PMC6071013; doi:10.3390/cells7070069)
Supplement: Supplementary file 1 [file cells-07-00069-s001.zip › Figure 6.pdf]

Figure 6

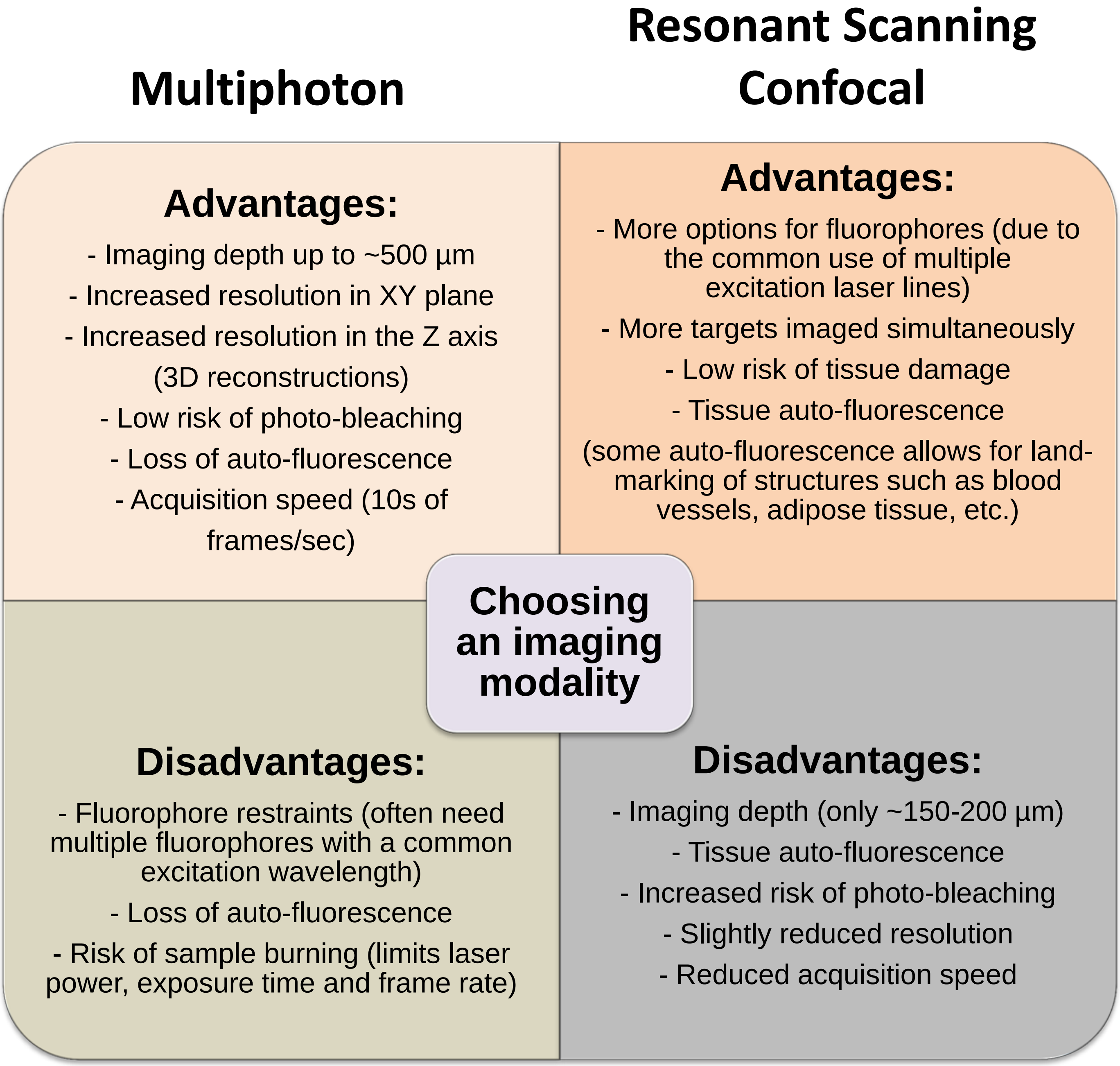

Figure 6. Comparing resonant scanning confocal versus multiphoton IVM imaging for studying cell recruitment and behaviour in the TME. Advantages and disadvantages of each imaging modality are listed to provide a basis for choosing which technique would best suit a specific experiment.
